# Updated secondary implant stability data of two dental implant systems. A retrospective cohort study

**DOI:** 10.4317/jced.54146

**Published:** 2017-09-01

**Authors:** Nicolas Grognard, Gino Verleye, Dimitrios Mavreas, Bart Vande-Vannet

**Affiliations:** 1 Kliniek Royal, Oostende, Belgium; PhD student @ CHIR- Unit Dentistry – ORHE, department of Orthodontics, Faculty of Medicine and Pharmacy, Vrije Universiteit Brussel, Belgium; 2 Professor, Communication Sciences, Universiteit Gent, Belgium; 3 Professor, CHIR- Unit Dentistry – ORHE, department of Orthodontics, Faculty of Medicine and Pharmacy, Vrije Universiteit Brussel, Belgium

## Abstract

**Background:**

At present, updated secondary implant stability data generated by actual versions of resonance frequency analysis (RFA) and mobility measurement (MM) electronic devices of 2 different implant systems with actual manufactured surfaces seem to lack and/or are incomplete.

**Material and Methods:**

Secondary implant stability data based on both RFA and MM measurements were collected and analyzed from 44 formerly treated patients (24 f, 20 m) that received either Ankylos Cellplus (Ø3.5mm) (A) (n=36) or Straumann regular neck SLA tissue level (Ø4.1mm) (S) (n=37) implants in posterior positions of both jawbones (total number= 72). These results were interpretated in view of formerly published data.

**Results:**

Estimated RFA outcomes (mean±SD) for A implants were of 81.23 (±0.65) (LP) - 76.15 (±1.57) (UP) isq; for S implants 76.15 (±1.48) (LP) - 73.88 (±2.34) (UP) isq. Estimated MM outcomes for A implants were (-4.0) (±0.23) (LP) - (-3.2) (±0.33) (UP) ptv; for S implants (-5.15) (±0.39) (LP) - (-4.4) (±0.84) (UP) ptv. According to GEE statistical modelling, implant type and – position seems to influence the outcome variables (*p*<0.05), gender and implant length did not (*p* >0.05).

**Conclusions:**

Secondary implant stability values, recorded with current RFA and MM devices, of A Cellplus implants are provided for the first time. A difference of 14.7-9.7 isq values was noted for CellPlus versus TPS S implants recorded with a cabled RFA device. This study supports the assumption that RFA outcomes generated with first generation RFA devices are different from those obtained with current RFA devices, meaning that its use in reviews need caution and correction.

** Key words:**Secondary implant stability, resonance frequency analysis, Periotest, Osstell Mentor, Straumann, Ankylos, CellPlus, SLA.

## Introduction

The biologic process occuring during uneventful osseointegration of oral implants follows a distinct and predictable biological pathway that has been demonstrated by several authors (1,2). Primary stability refers to the stability status of an implant immediately after insertion, whereas secondary stability refers to the stability status after completion of active osseointegration (3). Primary implant stability is a merely mechanical issue that is dictated by factors such as bone density, surgical bed preparation (under – or overpreparation), implant geometry (eg. cylindrical or tapered, non-self tapping or self tapping), implant length and - diameter (4-8). Subsequently, secondary stability is reached following the formation of new woven and lamellar bone onto the implant surface. With the advent and introduction of so-called ‘moderately’ rough surfaces, shortening of the healing period to 6-8 weeks was proposed for a the Straumann implant system specific implant system (9-11). Determination of implant stability makes part of several implant success criteria, both on long term (12) and short term (13,14). The latter author included to his set of success criteria already a notion of implant stability assessed by electronic devices as an alternative to manual tapping.

While insertion torque recording devices, either analogue or electronic, allow quantitative recording of a particular aspect of primary implant stability, RFA and MM recording devices are at present instruments that allow monitoring of the implant stability evolution during the osseointegration period at a level that is not feasible with traditional clinical or radiographical methods (15). Both the RFA and MM procedures involve as common working principle, measurement of lateral displacement and/or deflection of the implant in the surrounding bone after controlled stimulus application. In case of the Periotest method, the excitation effect is induced by controlled mechanical tapping. From insertion untill prosthetic loading, a healing abutment can serve as the transducer for the tapping device force application (16). RFA methdology is based on quantitative assessment of (micro)deflection of the implant induced by controlled appliance of electromagnetic excitation, by aid as an implant system specific transducer (17,18). Since the introduction of this technology, several generations of devices and transducers have been commercialized (19). The outcome of both RFA and MM methods is influenced by multiple factors. The properties of the tranducer (eg. stiffness and screw properties) , the stiffness of the ’implant-transducer’ complex , the properties and stiffness of ‘implant-bone’ complex, eg. influenced by the effective heigth of the coronal implant part above the bone crest and the implant surface texture (19), and the stiffness of the bone itself are measurement influencing factors (20). As both implant system surfaces and measurement devices properties change over time, updating implant stability seems of value. Differences in implant stability between various generations of specific implant systems with different surface characteristics are described (8-10). Besides, significant implant stability differences recorded by various generations of resonance frequency analysis (RFA) devices are published (21). Furthermore, RFA and/or mobility measuring damping capacity (MM) based implant stability data of some actual surface textured implant systems for specific implant diameters and/or positions are lacking. Objective measurement of secondary implant stability may allow the clinician to make the correct decision when to load an implant of a given type, and to make choices on a patient -to-patient basis as to the most advantageous protocol. To be able to do this, knowledge of values or value-ranges obtained by actual versions of the test devices after testing of actual implant that can serve as accepted normal outcomes and differences between various implant systems can be of help when interpretating individual implant measurements. At present, only a report of Ankylos TPS surfaced implants is available based on recordings by the previous cabled Osstell device (8). Furthermore, MM values for Straumann SLA surfaced implants were apparently not found in the literature. The aims of this clinical retrospective study were: 1: to report secondary implant stability data of Ankylos Cell Plus and Straumann tissue level SLA surfaced implants implants recorded by the Osstell Mentor and the Periotest device, 2: to explore effects on the secondary implant stability outcome measurements of secondary variables, 3: to interpretate these results in view of previous reports. For both implant systems, data were retrieved from files of formerly treated patients from specific anatomical regions that received a specific implant diameter for each implant system.

## Material and Methods

-Patient selection

This retrospective cohort study was conducted in a private periodontal practice. Data were retrieved from files of formerly treated patients. These patients were referred for the surgical part of implant therapy by their general dentist. The choice of the applied implant system was made by the general dentist. No data concerning primary stability or bone quality were present for analysis.

The following search criteria were adopted for case selection:

• Implant system specifications: Ankylos Cell Plus implants with a diameter of 3.5mm (A) (Dentsply Implants, Mannheim, Germany) or Straumann SLA tissue level RN with a diameter of 3.5mm.

• Transmucosal healing after implant insertion by aid of a healing abutment.

(A implants: Balance base posterior sulcus former , 3mm height (Dentsply Implants, Mannheim, Germany); S implants: Healing cap, 3mm height (Straumann AG, Basel, Switzerland))

• Insertion in posterior sites of the maxillary or mandibular jaw bone

• No previous or concommitant bone augmentation procedures.

• Uneventfull healing in the period between insertion and time of measurement.

• Normal clinical and radiographical aspects at the time of measurement.

• Avalailability of both RFA and MM data, recorded after a healing period of 8-12 weeks.

The search selection resulted in a a cohort of 44 formerly treated patients (22 females, 20 males) (mean age 56.2± 12.3 years; range: 34-78 years) ( Table 1). On implant level the cohort consisted of 73 implants (36 A implants, 37 S implants) with the following distribution: upper posterior regions (UP):19 A and 18 S implants, lower posterior regions (LP): 17 A and 19 S implants.

**Table 1:**
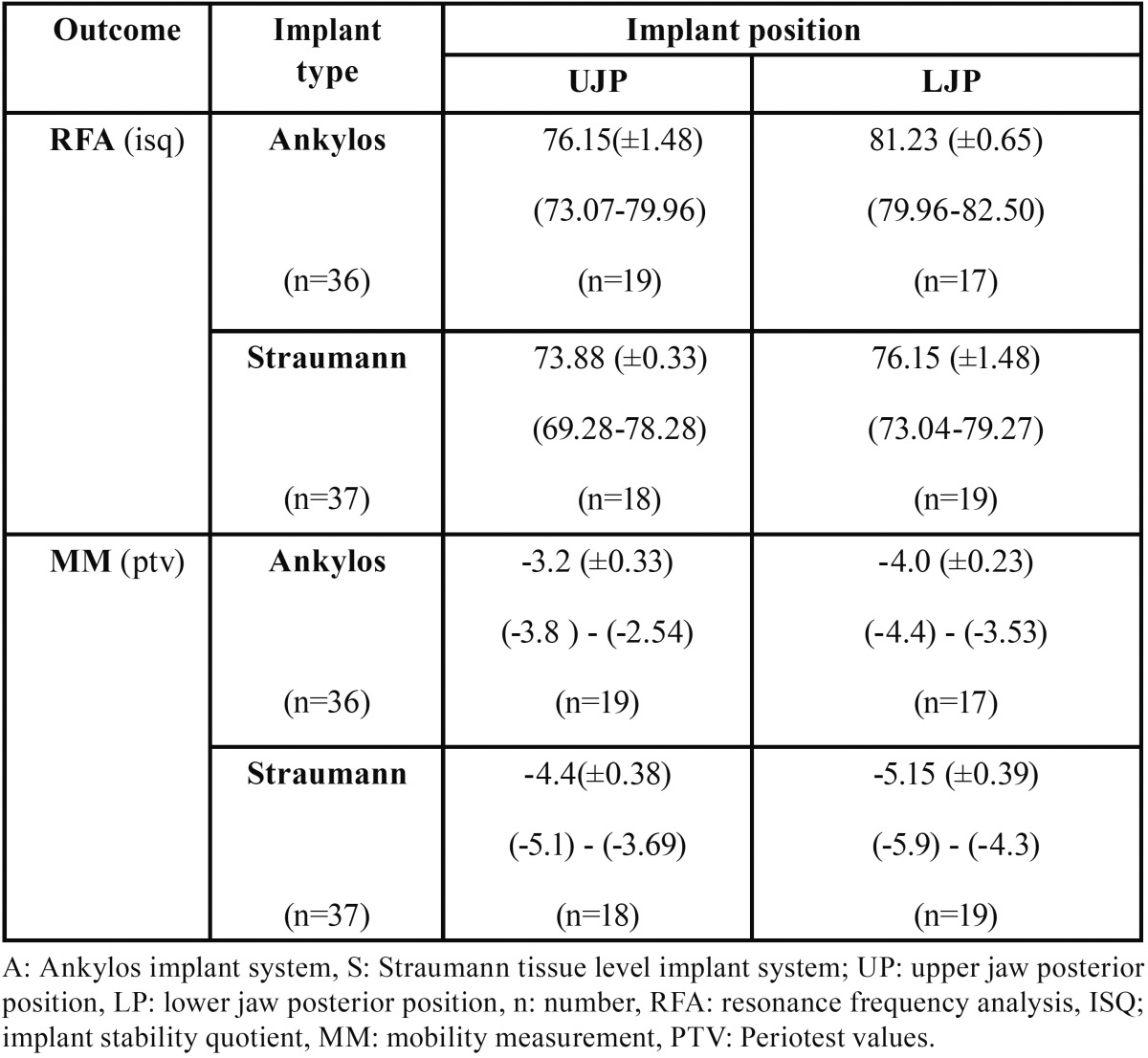
Secondary implant stability data mean (+/- SD) values and 95% CI’s according to implant type and implant position.

-Stability measurements

Implant stability data were collected after a mean healing period of approximately 8-10 weeks (A: LP: 8.7 ± 4.2 weeks, UP: 10.0 ± 4.3 weeks; S: LP: 8.0 ± 1.3 weeks, UP: 9.6 ± 4.1 weeks). Differences between groups regarding time of measurement were not significant different (data not shown). RFA measurements were performed on each implant at the implant level using a wireless type Osstell Mentor device (Osstell AB, Gothenburg, Sweden). An implant-specific transducer, called ‘Smartpeg’ (Osstell AB, Gothenburg, Sweden) was used for each implant type. For A implants, Smartpeg type 13 was used. For S implants, Smartpeg type 4 was used. RFA data are reported in implant stability quotient units (isq), wherein greater positive values indicate greater stability. MM measurements were performed at healing abutment level using a Periotest device (Gulden, Modautal, Germany). For both methodologies, measurements were repeated until a constant value was obtained. The last (consistently obtained) value was used for statistical analysis.

-Statistical analysis

The SPSS statistical software package 22.0 (IBM SPSS, Chicago, USA) was used. As approximately 33% of the patients received multiple implants, ‘Generalized Estimation Equation’ statistical modelling was applied. GEE was assumed to be appropriate to estimate parameters of a generalized linear model with a possible unknown correlation between outcomes (htpp://en.wikipedia.org/wiki/Generalized_estimating_equation). Mean secondary implant stability values were presented as estimates according to the GEE modelling with standard errors and 95% confidence intervals (CI). The effects of gender, implant type and implant length as dependent variables on the implant stability outcome was investigated after GEE modelling The null hypothesis of no effect in outcome for various both patient and implant related dependent variables was adopted. The level of significance was set at *p* = 0.05.

## Results

-Subject and implant distribution descriptive data 

Most retrieved cases received one (66.6 %) or two implants (20 %), 6 patients received 3 or more implants (13,2%) (data not shown). Most of the A implants placed in UP positions had an intra-bony length of 11 mm, whereas those placed in LP positions most commonly had an intra-bony length of 9.5 mm or 11 mm (data not shown). Most of the S implants placed in UP positions had an intra-bony length of 8 mm or 10 mm, whereas those placed in LP positions most commonly had an intra-bony length of 10 mm (data not shown).

-Secondary implant stability outcome

Overall mean implant (±SD) and 95% CI’s of secondary stability data for both RFA and MM testing of the two investigated implant implant systems are presented  Table 1. Both A and S implants exhibited higher and thus more favourable Osstell Mentor values in the mandible compared to the outcome in the maxilla. The same trend was observed for the MM outcomes for each implant system. According to the GEE modelling, a significant effect of implant position was observed on both RFA and MM outcomes ( Table 2). The effect of implant type was only significant on the RFA outcome, not on the MM outcome. Gender, implant length and healing time had no effect on the RFA and MM outcomes.

**Table 2:**
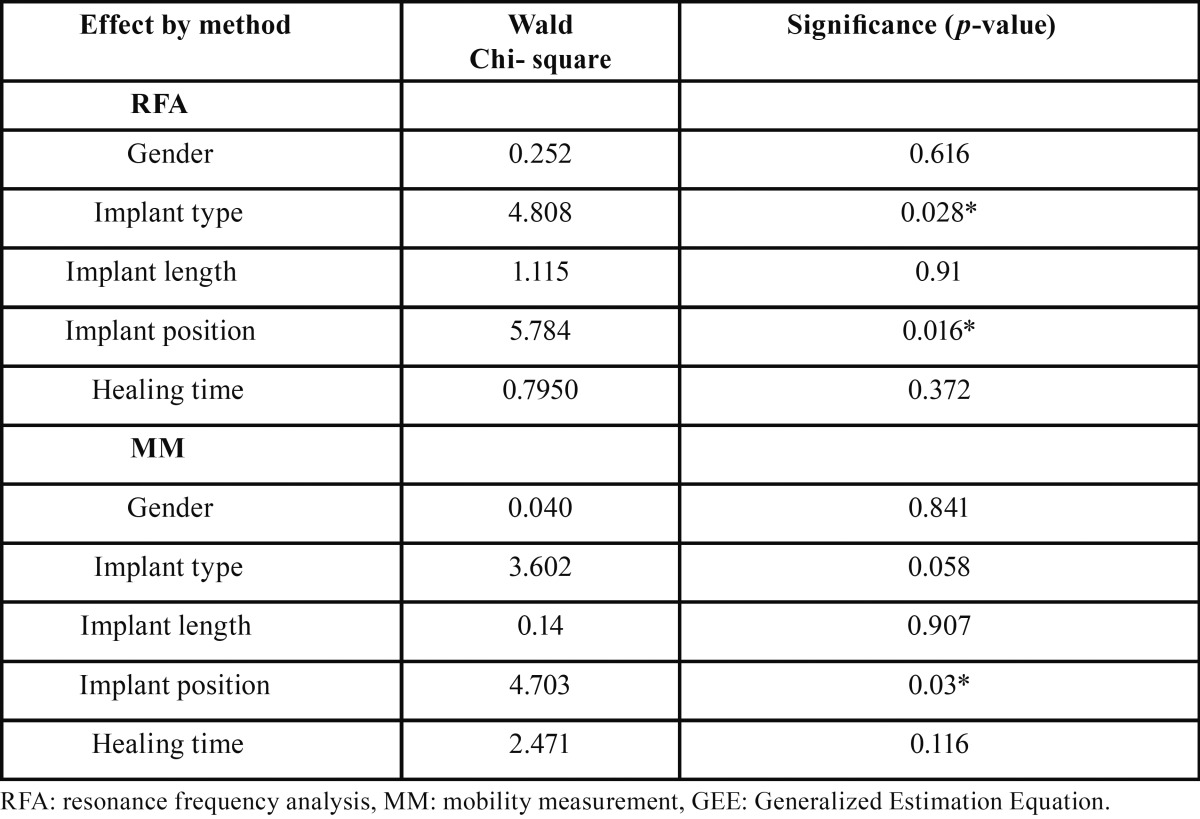
Effects of gender, implant type, implant length, implant position and healing time on secondary implant stability measurements according to GEE modelling.

## Discussion

In this study based on data retrospectively retrieved from patients referred for implant surgery, an attempt was made to report mean secondary implant stability values for two distinct implant types in two distinct areas of the oral cavity. All the implants were at the time of stability determination judged stable from a clinical and radiographical point of view, as part of the search criteria. One diameter per implant system was chosen for each implant system in order to exclude any influence of the latter on outcome variarables within groups. Likewise, only one inter-arch location was included to exclude any influence of whithin –arch variation. Beside, all MM measurements were performed at healing abutments with 3mm heigth in order to exclude measurement errors derived from variable striking point heights from the bony crest. Since many patients received multiple implants, data analysis was performed using GEE modelling to anticipate for possible unknown correlations between outcomes of multiple implants inserted in the same patient. Futhermore, the influence of patient and implant related variables on both outcome variables was explored .

As outlined in various guideline sets, the determination of implant stability or ‘implant immobility’ at various stages after implant insertion is considered of key importance in therapeutic decision-making (13-15). Several methodologies and/or tests to assess implant stability are described. Whereas some of them are applicable at multiple time-points after implant insertion, others has a more limited use, eg. determination of insertion torque (22). Furthermore, the application of removal torque can be questioned from an ethical point of view (23,24). Electronic devices such as RFA and MM damping capacity recording instruments possess the power to detect implant stability at the level that is not achieveable with traditional radiographical and/or clinical methods (3,14,15). For clinical application of both measurement systems, a transducer, meaning an implant system specific component, is needed to perform the measurement before prothetic loading: a healing abutment in case of MM and a Smartpeg in case of RFA. Both electronic devices applied in this retrospective study possess a substantial long time of use in implant dentistry. For Perio-test, changes over time only apply to changes of the housing hardware and no changes to measurement technology, meaning that over time, outcomes of various generations of MM devices do not differ when applied to a given implant system in comparable conditions (http://www.med-gulden.com). For Osstell RFA devices, changes over time do not only apply to the housing of the hardware but also to the intrinsic measurement technology but also to the data processing software (3). This means that differences of the application of various generations of RFA devices to identical implant systems in comparable conditions are to expected. The original version of the Osstell RFA device consisted of a wired version of the transducer. The transducer consisted of 2 built-in piezoceramic elements to be coupled to an individual implant, transmitting both the emitting signal and the captured evoked response. One piezoceramic element served as the transmittor element, receiving an electrically generated sine wave with varying frequency. The other piezoceramic element served as the receiver element capturing the induced vibrations in this spectrum and enabled a determination of resonance frequencies of the investigated implant. A rather practical shortcoming of this version what the fact that measurements had to be performed at 36 positions angled by 10° , thus covering a full spectrum of 360°. Meaning that the rather voluminous implant specific tranducer had to be (re)positioned every 10°. In this original approach the resonance frequency outcome was plotted against orientation. The response signal was analyzed by an oscilloscope with the resonance frequency in kHz as the outcome unit. The launch of the Osstell Mentor in 2004 included the introduction of a less voluminous, much more user-friendly, non-cabled transducer, called ‘Smartpeg’. Smartpegs are small aluminum rods with 3 different parts: a coronal part with an implant system specific screw fitting into the individual implant, a hexagon part enabling easy tightening / un-tightening and a magnet serving as the electromagnetic puls captor. The apparatus itself was a compact device with an incorporated microcomputer and electromagnetic signal emitting and receiving tipped probe. Excitation of the implant mounted Smartpeg is performed by 4 electromagnetic pulses with different frequencies inducing Smartpeg vibration in mostly 2 directions perpendicular to each other. The vibration directions correspond to a low and a high resonace frequency. The manufacturer recommends to perform at least 2 measurements, in order to identify these possible different stabilities. Furthermore, in order to suppress electromagnetic environmental noise, the working principle is refined by four times repeated emission of each excitation frequency. In summary, 16 pulses are emitted for each single measurements (20). The captured outcome of each in four fold emmitted signal is converted by the built-in microcomputer into a frequency spectrum by a ‘Fast Fourrier Transformation’ (FFT) method, ending up to detect among the 4 calculated spectra the 2 highest peaks representing the resonance frequencies of the implant. The latter will be used to calculate the so-called implant stability quotients (isq) by aid of a mathematical algorithm, which is Osstell Company ‘house secret’. The isq output is a unitless number, ranging between 0 and 100.

The manufacturers of both Osstell and Periotest devices provide in the web retrieveable manual ranges of values to which individual implant stability outcomes can be compared. In case of Periotest, a guide of outcome values ranges is proposed (http://www.med-gulden.com). A range from 0 to -8 ptv indicate good osseointegration and allow for prosthetic loading. A range from +1 to +9 indicate a warning that osseointegration might not be sufficient and a contra-indication for prosthetic loading. A range between +10 - +50 indicate clearly insufficient osseointegration and a contra-indication for loading. In case of Osstell, the listed isq ranges are the following: isq values <60 indicate an implant at risk, a range of 60-65 isq calls for traditional loading, a range of 65-70 isq calls for early loading and implants exhibiting isq values >70 isq can be cleared for immediate loading (http://www.osstell.com/clinicalguidelines). In these guidelines, the RFA device manufacturer does not comment how these ranges connect to a proper generation of used RFA device nor if corrections for outcome values obtained by first generations RFA devices need to be made. Within the limits of this retrospective study, updated secondary implant stability data based on RFA and MM measurements of different implant systems are reported. According to the GEE statistical modelling, implant type and – position seem to be influenced the outcome variables (*p*<0.05), gender and implant length did not (*p*>0.05).

When comparing the estimated secondary stability data based on RFA data reported in this retrospective study, the ranges of A implants: 76.15 ±1.51 isq (UJP) – 81.23 ±0.65isq (LJP), and the S implants: 73.88 ±2.34 isq (UJP) – 76.15 ±1.48 isq (LJP), exceed by large the range 60-65 isq in case of traditional loading proposed in the Ostell guidelines. When comparing the secondary stability data based on MM data reported in this retrospective study, the ranges of A implants: -3.2 ± 0.33 ptv (UJP) – -4.0 ±0.23 ptv (LJP), and the S implants: -4.4 ±0.38 ptv (UJP) – -5.15 ±0.39 ptv (LJP), situate perfect in the range of 0 to -8 in case of traditional loading proposed in the Gulden guidelines. To interpretate the present data, publications reporting implant secondary stability values for the presently described implant implant systems and surfaces were searched. Publications were whithheld that provided short term longitudinal evolution of implant stability following insertion in posterior areas of the oral cavity with implant characteristics quite comparable to those in the present study (10,25-29). Surprisingly, detailed, prospective study information was only availaible for implant stability analysed by RFA device for the Straumann implant system ( Table 3) and not for A CellPlus textured implant system. Viceversa, MM derived data were found only for the A implant (30,31) and not for the S implant system.

**Table 3:**
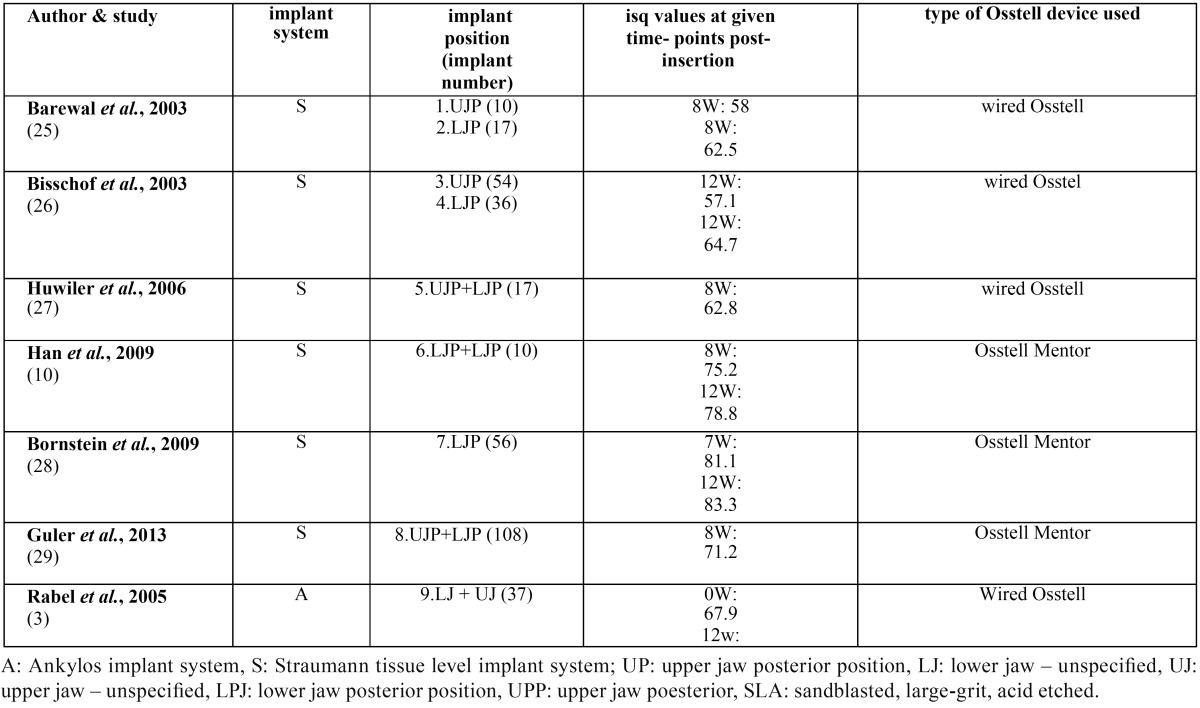
Published RFA based secondary implant stability data (mean values) for Straumann tissue level RN SLA surfaced implants (diameter=4.1mm) and Ankylos TPS surfaced implants (combined diameters).

The estimated MM values in the present study for the Cell Plus textured A implant system, (-4.0) (±0.23) ptv (LP) - (-3.2) (±0.33) ptv (UP), seem comparable with the reported ones for the TPS textured A system (30,31) (data not shown).

The estimated RFA outcomes for the Straumann implants in the present study seems to be well in line with the results in the above mentioned studies (10,28,29). In these studies the wireless Osstell device was used to assess RFA based outcomes for Straumann tissue level implants with a diameter of 4.1mm. For combined UP and LP positions a range of 71.2 – 75.2 isq was noted after recording with the Osstell Mentor device after 8 weeks (10,29), in one study (28) the come for LP positions was 81.1 isq after 7 weeks. In our study, an RFA outcome of 76,1 isq was noted for LP positions. When comparing the ranges of secondary implant stability of Straumann tissue level SLA Ø 4.1mm implants beween recording with the wired and wireless RFA device versions, a magnitude of difference of 8.4-18.6 isq units can be noted. According to the latter studies, in case of uneventfull healing of the Straumann system, the ‘stability dip’ was consistently described between two and four weeks (10,28,29). Thus, single stability measurements after 8-12 weeks seem to be clinically meaningfull in the course of uneventfull healing during the osseointegration period. In an *in vitro* approach (21), representing primary stability, compared the RFA outcome of MIS implants with diameters 3.75mm and 4.2mm (MIS Implants Technologies Ltd., Shlomi, Israel), inserted in cadaver jaw bone between the cabled Osstell device with the Osstell Mentor version. The mean outcome for the 3.75mm Ø was 41.6 (±9) isq for the cabled version versus 52.7 (±9) isq mm for the wireless version, indicating a difference of 11 isq. The mean outcome for the 4.2mm Ø was 50.9 (±9) isq for the cabled version versus 62.9 (±7) isq for the wireless version, indicating a difference of 12 isq. It can be concluded that for at least 3 implant systems, major and clinical important differences exist in outcome between cabled and non-cabled versions of Osstell devices.

 To interpretate specifically the RFA based secondary implant stability data reported in this study for Cellplus A implants, the data provided in the only single report by Rabel *et al.* (8) were used for comparing. The investigated Ankylos implant type in this study concerned the original commercialized Ankylos implant type characterized by a machined collar and a TPS surface. The currently available Ankylos implant type is characterisized by a moderately rough titanium surface along the full implant body without machined collar. In this study, both primary and secondary implant stability values of 37 A TPS implants recorded by aid of the cabled version of the RFA device and an F 23 type wired transducer were analyzed. In this analysis however no clear description of implant diameter and/or insertion position was provided. For the total sample of 37 implants, a mean RFA outcome of primary implant stability of 67.9 isq and 66.5 isq for secondary implant stability was described (sd not provided). A particular finding in this study was the fact that the mean primary implant stability value was larger than the mean secondary stability value for this particular implant system. The authors stated that this result could be explained that the insertion torque value for this non-self tapping implant system was extentively higher compared to the other self tapping system analyzed in this particular study. The RFA outcomes concerning secondary implant stability values of A CellPlus implants in this study ranged between 76.2 (±1.6) isq for UP positions and 81.2 (±1.6) isq for LP positions. When subtracting this estimates range from the mean value provided by Rabel *et al.*, a magnitude of difference range of 9.7-14.7 isq values is obtained.

Within the limits of this study, we assume that the outcomes reported for A CellPlus surfaced implants may serve as a reference to which secondary implant stability outcomes of individual implants can be compared in conventional loading protocols after an uneventfull healing period of 8-12 weeks. Hereby, implant type and – position seem to be influenced the outcome variables (*p*<0.05), gender and implant length did not (*p*>0.05), ( Table 4).

**Table 4:**
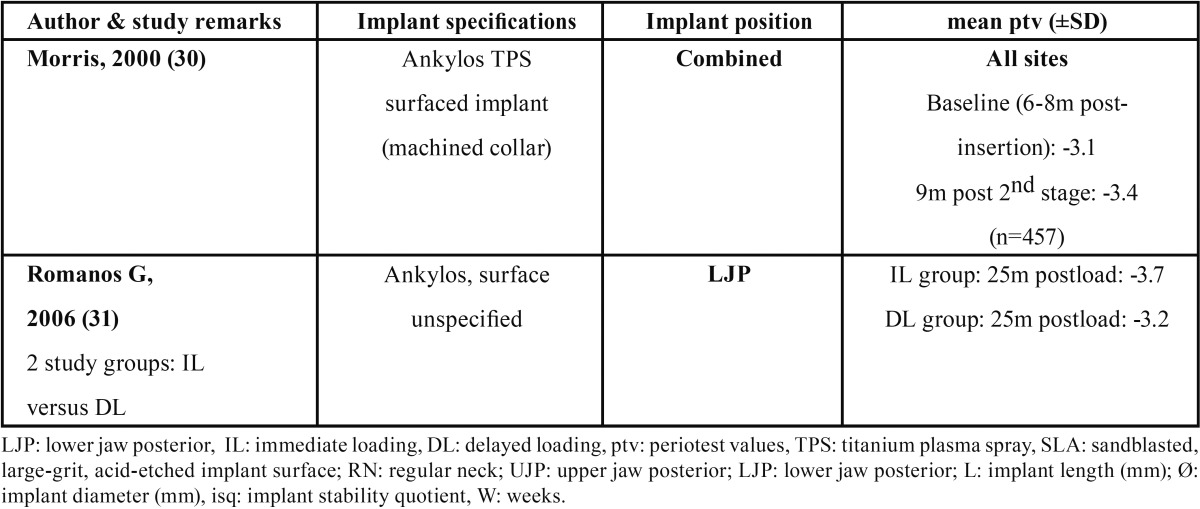
Published MM based secondary implant stability values (mean values) for Ankylos implants (combined diameters).

## Conclusions

When comparing the secondary implant stability outcome of A CellPlus implants reported values of A TPS implants recorded by the cabled Osstell device, a difference of 9.7-14.7 isq values was noted. The magnitude of this difference is comparable with the described differences in clinical studies for S tissue level implants after recording with the cabled and wireless RFA devices and also for MIS implants in an in vitro experiment. The magnitude of the RFA outcome difference between both generations of the Osstell device is clinically significant. This implies that the use of data derived after recording with different generations of RFA devices in reviews should be done with caution and need for correction.
